# Effect of Al on the Oxidation Behavior of TiCrZrNbTa High-Entropy Coatings on Zr Alloy

**DOI:** 10.3390/ma18091997

**Published:** 2025-04-28

**Authors:** Min Guo, Chaoyang Chen, Bin Song, Junhong Guo, Junhua Hu, Guoqin Cao

**Affiliations:** 1 Lonemen Laboratory, Luoyang 471000, China; g19960413mbs@163.com (M.G.); 13283071567@163.com (C.C.); songbinzzu@163.com (B.S.); 15835436647@163.com (J.G.); 15238209837@163.com (J.H.); 2 School of Materials Science and Engineering, Zhengzhou University, Zhengzhou 450001, China

**Keywords:** refractory high-entropy alloy coatings, aluminum alloying, protective oxide layer, interfacial bonding, accident-tolerant fuel cladding

## Abstract

This study investigates the role of Al alloying in tailoring the oxidation resistance of AlTiCrZrNbTa refractory high-entropy alloy (RHEA) coatings on Zry-4 substrates under high-temperature steam environments. Coatings with varying Al contents (0–25 at.%) were deposited via magnetron sputtering and subjected to oxidation tests at 1000–1100 °C. The results demonstrate that Al content critically governs oxidation kinetics and coating integrity. The optimal performance was achieved at 10 at.% Al, above which a dense, continuous composite oxide layer (Al_2_O_3_, TiO_2_, Cr_2_O_3_) formed, effectively suppressing oxygen penetration and maintaining strong interfacial adhesion. Indentation tests confirmed enhanced mechanical integrity in Al-10 coatings, with minimal cracking post-oxidation. Excessive Al alloying (≥17 at.%) led to accelerated coating oxidation. This work establishes a critical Al threshold for balancing oxidation and interfacial bonding, providing a design strategy for developing accident-tolerant fuel cladding coatings.

## 1. Introduction

Due to the Fukushima nuclear accident, public concerns about nuclear safety have increased, significantly impacting the global nuclear power industry [1,2,3]. In response, the international community has been dedicated to developing a new type of nuclear fuel capable of withstanding similar accidents, known as accident-tolerant fuel (ATF) [4,5]. One potential approach is the application of protective ATF coatings on the surface of fuel cladding [6]. Various candidate coating materials and deposition techniques have been explored for zirconium alloy fuel cladding. Current research on fuel cladding coatings mainly includes metal coatings [7,8,9,10] and ceramic coatings [11,12,13,14,15]. Tang et al. [14] compared the performance of different coatings under both normal operating conditions (subcritical water) and accident conditions (high-temperature steam). Al- and Si-rich coatings exhibited promising behavior under accident conditions but performed poorly under normal conditions. Ti- and Ni-based coatings failed to adapt to high-temperature steam, whereas Cr-based coatings demonstrated superior performance under both conditions, making them a promising choice for cladding materials. However, at elevated temperatures, elemental interdiffusion between Cr coatings and Zr alloy substrates leads to premature coating failure. Thus, further optimization of coating compositions is necessary to enhance their resistance to extreme conditions.

High-entropy alloys (HEAs) exhibit unique properties, including high hardness, wear resistance, corrosion resistance, and thermal stability, due to high-entropy effects, lattice distortion, sluggish diffusion, and the “cocktail effect” [16,17,18,19]. These properties make HEAs highly promising for surface protection applications. To ensure suitability for extreme environments such as aerospace and nuclear reactors, new alloys should be composed of high-melting-point elements. Based on HEA concepts, Senkov et al. [20] introduced the concept of refractory high-entropy alloys (RHEAs), which differ from traditional HEAs by incorporating refractory metals instead of transition metals. RHEAs exhibit excellent mechanical properties [21,22,23] and radiation resistance [24,25,26,27]. However, due to the high oxygen affinity of refractory metals, severe oxidation occurs at elevated temperatures [28,29,30,31]. To counteract this, researchers have explored the addition of elements such as Al and Si to improve the oxidation resistance. Al_2_O_3_ layers can reduce oxidation kinetics, while Si prevents the formation of volatile vanadium oxides and promotes the development of a stable oxide layer while minimizing nitridation [28,29,30,31]. Anber et al. [29] investigated the effect of Al addition (4.8 and 13 at.%) on HfNbTaTiZr alloys and conducted oxidation tests at 900 °C. The results showed that Al significantly reduced the oxidation weight gain rate. Similarly, Pei et al. [30] studied the impact of Al on the oxidation resistance of TiZrV_0.5_Nb_0.5_ RHEAs, preparing TiZrV_0.5_Nb_0.5_, TiZrV_0.5_Nb_0.5_Si_0.3_, and TiZrV_0.5_Nb_0.5_Al_0.7_ alloys. Oxidation experiments at 1000 °C revealed that all samples exhibited improved oxidation resistance, with TiZrV_0.5_Nb_0.5_Al_0.7_ forming an Al_2_O_3_ protective layer. The dense oxide structure inhibited extensive inward oxygen diffusion, thus preventing severe internal oxidation. The low oxygen diffusion rate also suppressed the formation of volatile oxides, thereby preventing oxide layer cracking.

Given the safety concerns associated with traditional zirconium alloys, there is an urgent need to develop high-performance coatings. This study combines the high-temperature oxidation resistance of Cr-based coatings with the superior properties of RHEAs. Ti, Cr, Zr, Nb, and Ta were selected as refractory metals to provide excellent mechanical properties and high-temperature stability, while Al was incorporated to enhance oxidation resistance. Using magnetron sputtering, TiCrZrNbTa coatings with varying Al contents were deposited onto Zry-4 alloy substrates. High-temperature steam oxidation tests were conducted at 1000–1100 °C to evaluate the effect of Al content on oxidation resistance. The optimal coating composition was identified, and the morphology, structure, and element diffusion behavior of the oxidized coatings were analyzed in detail.

## 2. Experimental Details

A magnetron sputtering system was used to deposit refractory high-entropy alloy (RHEA) coatings onto the zircaloy-4(Zry-4) substrates. The chemical composition of the Zry-4 is Zr-98.2%, Sn-1.5%, Fe-0.2%, and Cr-0.1%. A circular target with a diameter of 76.2 mm was composed of sector-shaped metallic targets of Al, Ti, Cr, Zr, Nb, and Ta, positioned at different angles. The sources of Zry-4 and metallic targets were made by (ZhongNuo Advanced Material Technology Co., Ltd., Beijing, China). The AlTiCrZrNbTa coatings were deposited onto polished Zry-4 alloy substrates using direct current (DC) magnetron sputtering. The composition of the coatings was adjusted by varying the proportions of target materials based on their respective sputtering yields, while the sputtering current and power were fine-tuned to control the final coating composition.

A tube furnace was preheated to the target temperatures (1000 °C and 1100 °C) prior to placing the coated samples inside for the isothermal experiment. A steam environment was established by using ultrapure water as the steam source. Steam was introduced at a flow rate of 0.2 mL/min after the furnace reached 150 °C. The high-temperature steam oxidation experiments were conducted by exposing the samples to 1000 °C for 60 min and 1100 °C for 30 min in the furnace, followed by air cooling. The thickness of the as-deposited coatings was measured using a Dektak XT profilometer (Shanghai R&D Instrument Co., Ltd., Shanghai, China). To minimize measurement error, each sample was measured three times, and the average value was taken as the final thickness.

Mechanical tests were conducted on the oxidized coatings. A microhardness tester equipped with a square-shaped indenter was used. The applied load was set to 0.98 N, with a dwell time of 15 s. The indentation size was in the micron range, allowing for an overall evaluation of the mechanical properties across the coating thickness. The coated samples were weighed before and after oxidation at various time intervals using an electronic balance. To reduce measurement error, each sample was weighed at least three times.

The macroscopic morphology of the coatings after high-temperature steam oxidation was characterized using an optical microscope (OM) from Zeiss, Oberkochen, Germany (Axio Scope A1 Pol). The cross-sectional samples were ground and polished sequentially using SiC sandpapers with grit sizes of 100–2000#, followed by fine polishing with W2.5 μm and W0.5 μm diamond suspensions. The phase composition of the coatings was analyzed using an X-ray diffraction (XRD) system (Empyrean, PANalytical, Almelo, The Netherlands) with a Cu target as the radiation source. Measurements were performed using the grazing incidence X-ray diffraction (GIXRD) mode with a parallel beam setup. A Helios G4 CX dual-beam scanning electron microscope (SEM) from Thermo Fisher Scientific (Brno, Czech Republic) was used to analyze the coating morphology. Both secondary electron (SE) and backscattered electron (BSE) modes were employed, with a resolution of ≤0.6 nm in SE mode and ≤2 nm in BSE mode at high voltage. Additionally, an energy-dispersive spectroscopy (EDS) system was integrated into the SEM to analyze elemental distribution and composition, providing insights into the oxidation resistance of the coatings.

## 3. Results and Discussion

AlTiCrZrNbTa coatings with varying Al content were deposited on Zry-4 alloy substrates using single-target magnetron sputtering. The compositions of the as-sputtered coatings are listed in Table 1. Based on Al content, the coatings were designated as Al-0, Al-5, Al-10, Al-17, and Al-25. All coatings maintained a consistent thickness of ~4 μm to eliminate thickness effects on oxidation behavior. The ideal mixing entropy was calculated by the following equation [32]:

$$\Delta S_{\mathrm{mix}}=-R\sum x_{i}\ln x_{i}$$

where R is the gas constant and $x_{i}$ represents the molar fraction of element i. Thermodynamic calculations revealed that increasing Al content enhanced the ideal mixing entropy from 1.46R to 1.73R, aligning with high-entropy alloy design principles.

**Figure 1:**
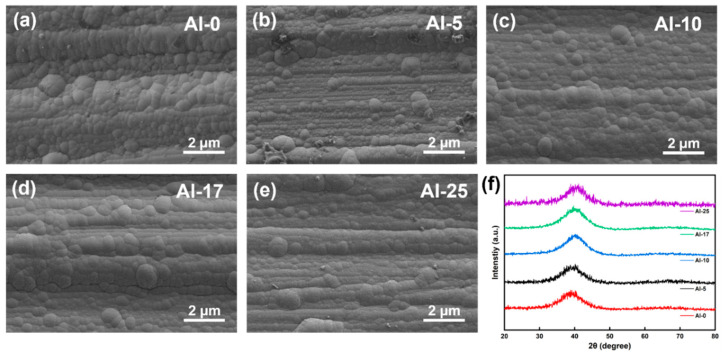
SEM images of the surfaces of AlTiCrZrNbTa coatings in sputtered states: (**a**) Al-0 coating; (**b**) Al-5 coating; (**c**) Al-10 coating; (**d**) Al-17 coating; (**e**) Al-25 coating; (**f**) XRD spectra of AlTiCrZrNbTa coatings in sputtered states.

Figure 1a–e display SEM surface images of the coatings, revealing dense and defect-free morphologies. XRD patterns in Figure 1f exhibit a broad diffraction peak, indicating an amorphous structure, consistent with previous reports on sputtered high-entropy alloy coatings [33,34]. Notably, Al incorporation induced gradual peak shifts toward higher angles, suggesting a denser atomic packing density.

**Figure 2:**
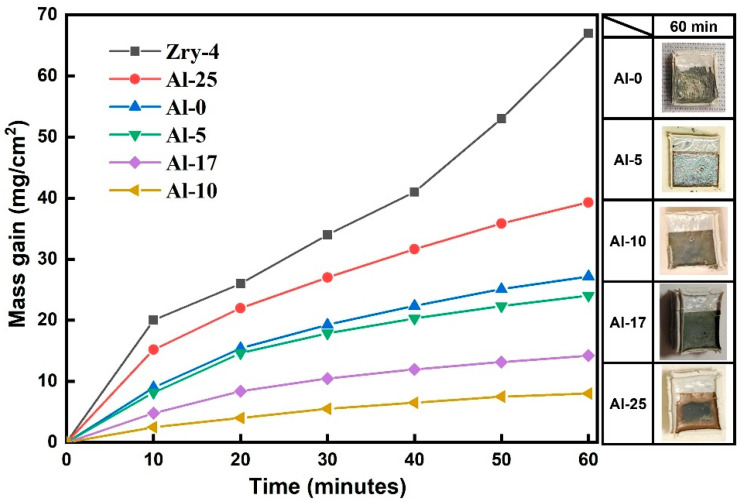
Specific mass change as a function of time for AlTiCrZrNbTa coatings during isothermal exposure to steam at 1000 °C and macroscopic morphology of the sample after oxidation.

To investigate the influence of Al content on the oxidation resistance of high-entropy alloy coatings against high-temperature steam, the oxidation experiment at 1000 °C was conducted on Zry-4 alloy with Al_x_TiCrZrNbTa (x = 0, 5, 10, 17, 25 at.%) coatings. In Figure 2, it can be observed that the oxidation weight gain of all coating systems follows a parabolic pattern. The parabolic rate constant, *k_p_*, was calculated by the mass gain per unit area of a sample (Δ*W/A*) and oxidation time (*t*) according to Equation [35]:

$${\left(\frac{∆W}{A}\right)}^{2}=k_{p}t$$

The parabolic rate constant, *k_p_*, is about 13.1, 10, 1.2, 3.5, 25.9 mg^2^ cm^−4^ min^−1^ for Al-0, Al-5, Al-10, Al-17, Al-25, respectively. In addition, a comparison of the *kₚ* values obtained in this work with literature data [35,36,37] indicates that the coatings fabricated in this study possess excellent resistance to high-temperature oxidation. The Al content significantly affects the oxidation kinetics behavior. Coatings with low Al content (x ≤ 5) have a higher oxidation rate, and the substrate undergoes significant deformation accompanied by large-scale coating delamination; when the Al content increases to 10 at.%, the oxidation weight gain is the lowest, and the coating maintains high integrity, indicating its good oxygen-blocking ability, while coatings with excessively high Al content (x ≥ 17) result in a further increase in oxidation weight gain, and the deformation of the substrate and coating delamination become more severe.

**Figure 3:**
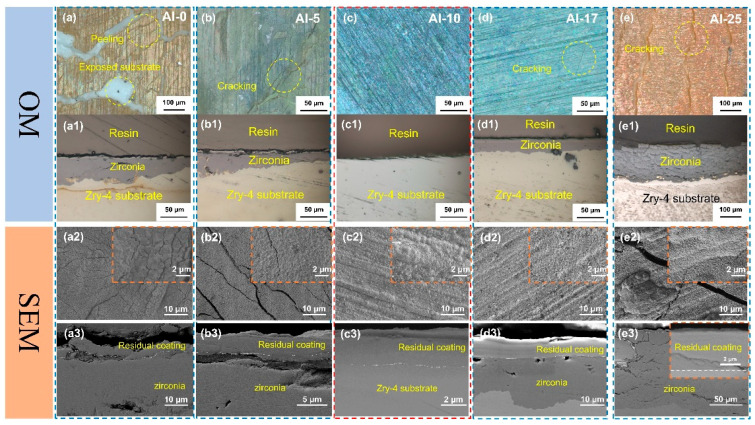
Surface and cross-sectional morphologies of Al-0, Al-5, Al-10, Al-17, and Al-25 coatings after oxidation at 1000 °C for 60 min: (**a**–**a3**) Al-0 coating; (**b**–**b3**) Al-5 coating; (**c**–**c3**) Al-10 coating; (**d**–**d3**) Al-17 coating; (**e**–**e3**) Al-25 coating.

Figure 3 shows the OM and SEM images of the surface and cross-section morphology after coating oxidation. From the OM images (Figure 3a–e,a1–e1), it can be observed that a large number of cracks appeared on the surfaces of Al-0, Al-5, Al-17, and Al-25 coatings, which provided channels for O to directly contact and react with the Zr matrix. However, the surface of the Al-10 coating remained relatively smooth after oxidation, without obvious macroscopic defects, which preliminarily verified its superior oxidation resistance. The statistical results of the experimental data are shown in Table 2. Only the Al-10 sample remained intact after high-temperature water vapor oxidation, and the coating effectively blocked the invasion of the O element, while the Al-0, Al-5, Al-17, and Al-25 coatings failed after long-term oxidation. The preliminary judgment indicates that an appropriate amount of Al alloying can improve the oxidation resistance of the coating, but when the Al content exceeds 15 at.%, the coating’s oxygen-blocking ability decreases.

A dense and continuous oxide layer can effectively prevent O from eroding the residual coating and the substrate [31]. From the SEM morphology analysis, the TiCrZrNbTa coating without Al alloying formed granular oxides after oxidation (Figure 3a2,a3), and the cross-sectional morphology further shows that the coating cracked and was not continuous after oxidation, and was completely separated from the substrate. The high-magnification images showed that the granular oxides were more densely combined for the Al-5 coating. However, at the macroscopic scale, there were still a large number of cracks on the coating surface. The cross-sectional images (Figure 3b3) indicated that the coating remained relatively continuously distributed on the substrate, but was still separated from the substrate. With further increase in Al content, the Al-10 coating demonstrated superior oxidation, and the surface was mainly composed of dense granular oxides (Figure 3c2). The cross-sectional morphology (Figure 3c3) showed that the coating exhibited strong bonding with the substrate, and no obvious cracks or defects were observed at the interface. The oxidized surface remained smooth for the Al-17 coating (Figure 3d2), but there were holes in local areas. The cross-sectional morphology (Figure 3d3) showed that the coating was intact without obvious cracks or defects and was closely bonded to the substrate. However, the oxidation of the Zr substrate was identified, indicating that the coating failed to form an effective oxygen-blocking structure. When the Al content further increased to 25 at.%, cracks reappeared on the oxidation surface of the coating (Figure 3e2). The cross-sectional image (Figure 3e3) shows that the substrate has been severely oxidized. However, the interfacial bonding between the coating and the substrate remains relatively dense.

**Figure 4:**
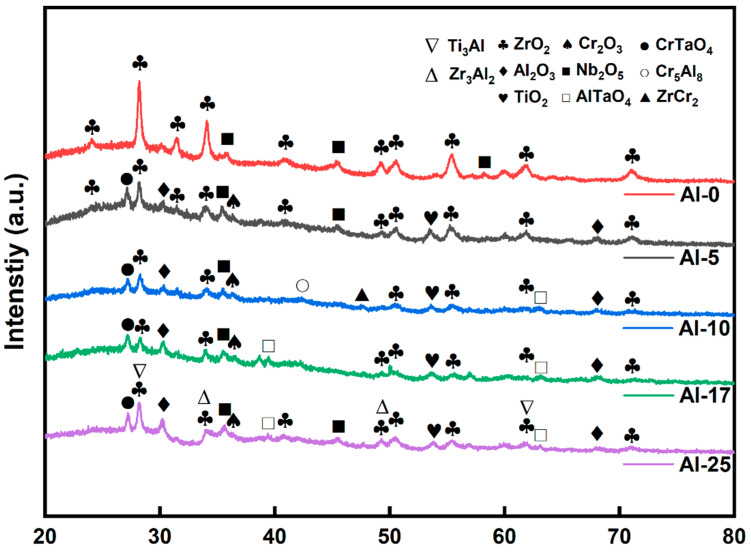
GIXRD spectra of the oxidized state AlTiCrZrNbTa coating.

Figure 4 shows the GIXRD diffraction patterns of the coated sample after oxidation. For Al-0 coating, ZrO_2_ and Nb_2_O_5_ were mainly detected, and the corresponding surface OM morphology showed that the coating had peeled off, causing the substrate to be directly exposed to the high-temperature water vapor environment and oxidized, thereby forming a large amount of ZrO_2_. In contrast, for the Al-5, Al-10, Al-17, and Al-25 coatings, due to higher integrity, in addition to detecting simple oxides such as Al_2_O_3_, TiO_2_, Cr_2_O_3_, ZrO_2_, and Nb_2_O_5_ in the diffraction patterns, CrTaO_4_ composite oxides were also observed. This phase has been reported to be formed by the further reaction of Cr_2_O_3_ and Ta_2_O_5_ [28]. With the increase in Al content, in the Al-17 and Al-25 coatings, AlTaO_4_ was further detected, indicating that a high Al content may promote the formation of complex oxides. In addition, in the Al-10 coating with superior oxygen-blocking performance, intermetallic compounds Cr_5_Al_8_ and ZrCr_2_ were identified, which may be related to the incomplete oxidation of the coating during the oxidation process.

**Figure 5:**
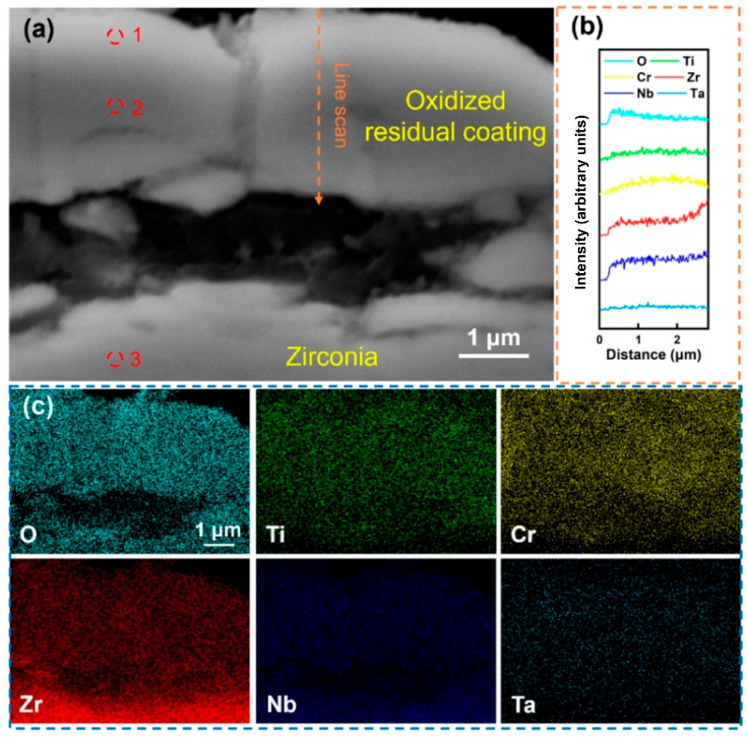
(**a**) Cross-sectional SEM images of the Al-0 coating after holding it in steam at 1000 °C for 60 min; (**b**) EDS line scan of (**a**) at the marked position; (**c**) EDS mapping of (**a**); the corresponding components in (**a**) are shown in Table 3.

Figure 5 shows the local magnified SEM morphology of the Al-0 coating after being held at 1000 °C in water vapor for 60 min. The results indicate that under the action of high-temperature water vapor, the coating was severely fragmented and completely separated from the substrate, and obvious cracks can also be observed in the independent remaining coating. The line scan in Figure 5b shows that the content of the O element gradually decreased from the outer layer to the inner layer of the coating, while other elements were relatively uniformly distributed within the coating area, and no significant enrichment was observed. The determination of the composition of different areas of the coating was carried out, and the result of site 1, shown in Table 3, indicates that the Cr content in the coating surface layer after oxidation was relatively high, indicating that Cr undergoes significant surface diffusion during oxidation. The composition analysis in site 2 shows that the content of the O element in this area was high, indicating that the coating has been completely oxidized. After removing the O element, the Cr content of the residual coating is lower than that of the deposited state coating due to surface diffusion, while the contents of Ti, Zr, Nb, and Ta change slightly, indicating that the diffusion behavior of these elements during oxidation is not obvious. In addition, the composition analysis result of site 3 shows that the substrate has been completely oxidized, and the coating fails to provide effective oxidation resistance protection. Overall, the RHEA coating without Al alloying undergoes severe fragmentation during high-temperature oxidation and cannot form a complete oxygen-blocking structure, resulting in the exposure of the substrate and intense oxidation.

**Figure 6:**
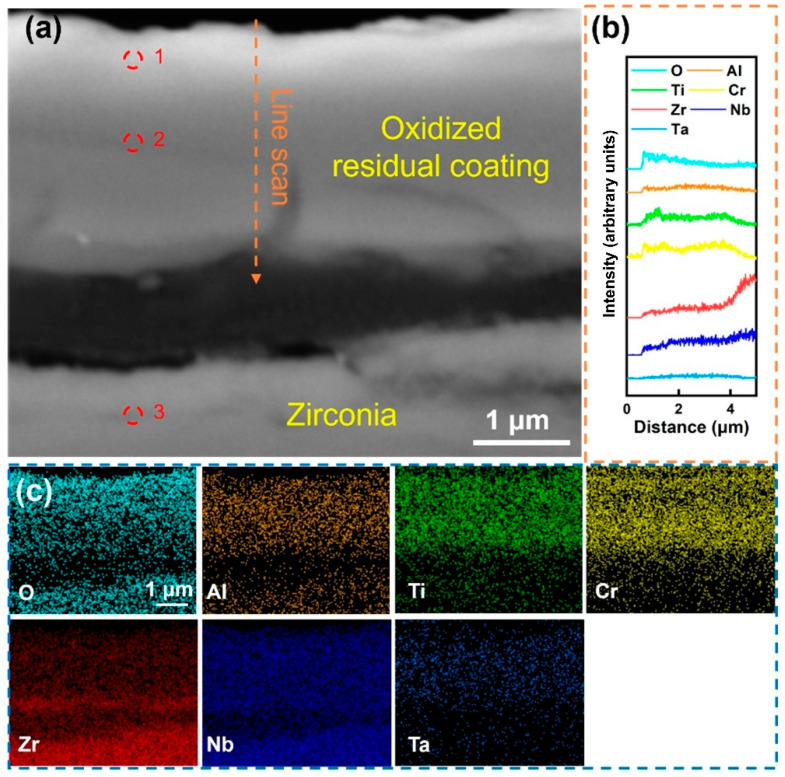
(**a**) Cross-sectional SEM images of the Al-5 coating after holding it in steam at 1000 °C for 60 min; (**b**) EDS line scan of the figure (**a**) at the marked position; (**c**) EDS mapping of (**a**); the corresponding components are shown in Table 4.

Figure 6a shows the local magnified morphology of the Al-5 coating after being kept in a water vapor environment at 1000 °C for 60 min. Compared with Al-0, the Al-5 coating maintained a complete structure and avoided severe cracking and peeling phenomena. The EDS analysis at site 1 in Table 4 confirms that the oxide layer was mainly composed of Cr_2_O_3_, accompanied by Al_2_O_3_ and TiO_2_ inclusions. This indicates that Cr underwent significant external diffusion, which was conducive to the formation of the oxide layer. Meanwhile, the element distribution within the coating was relatively uniform, and site 2 shows that the O content in this area is lower than that of the surface oxide layer, indicating that the formation of the surface oxide layer improves the oxygen-blocking ability of the coating to a certain extent. However, although the Al-5 coating can maintain good integrity, there are still certain issues with its bonding performance to the substrate. In addition, site 3 indicates that the matrix has undergone severe oxidation. After long-term oxidation, the coating and the substrate became seriously separated.

**Figure 7:**
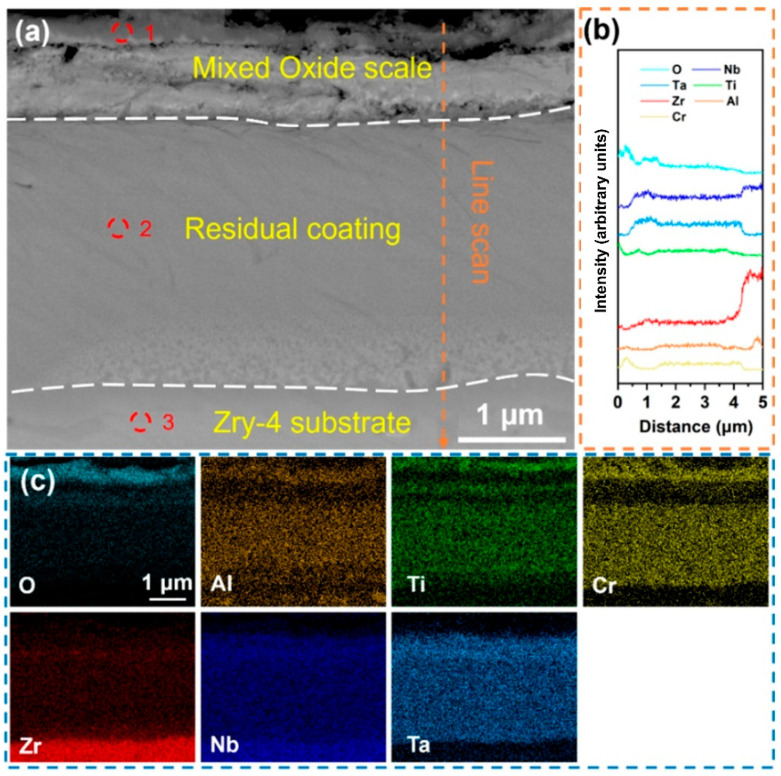
(**a**) Cross-sectional SEM images of the Al-10 coating after holding it in steam at 1000 °C for 60 min; (**b**) EDS line scan of (**a**) at the marked position; (**c**) EDS mapping of (**a**); the corresponding components in (**a**) are shown in Table 5.

Figure 7 displays the cross-sectional morphology and elemental distribution of the Al-10 coating after oxidation in a 1000 °C water vapor environment for 60 min. A surface oxide layer with high oxygen content was observed (Figure 7c), enriched with Al, Ti, and Cr. Elemental line scanning (Figure 7b) also confirmed significant surface enrichment of Al, Ti, and Cr, suggesting their active participation in oxide layer formation during oxidation. Compositional analysis (Table 5, Site 1) revealed that the oxide layer primarily consists of Cr_2_O_3_, alloyed with Al and Ti. The oxide layer exhibited a dense, defect-free interface with the underlying coating, indicating strong adhesion and uniform elemental distribution within the residual coating. Notably, the residual coating (Site 2) showed markedly reduced oxygen content compared to low-Al counterparts, demonstrating enhanced oxygen-blocking capability. Furthermore, negligible oxygen diffusion into the substrate (Figure 7c) and a significant decrease in matrix oxygen content (Site 3) confirmed the coating’s effective barrier against oxidative penetration.

**Figure 8:**
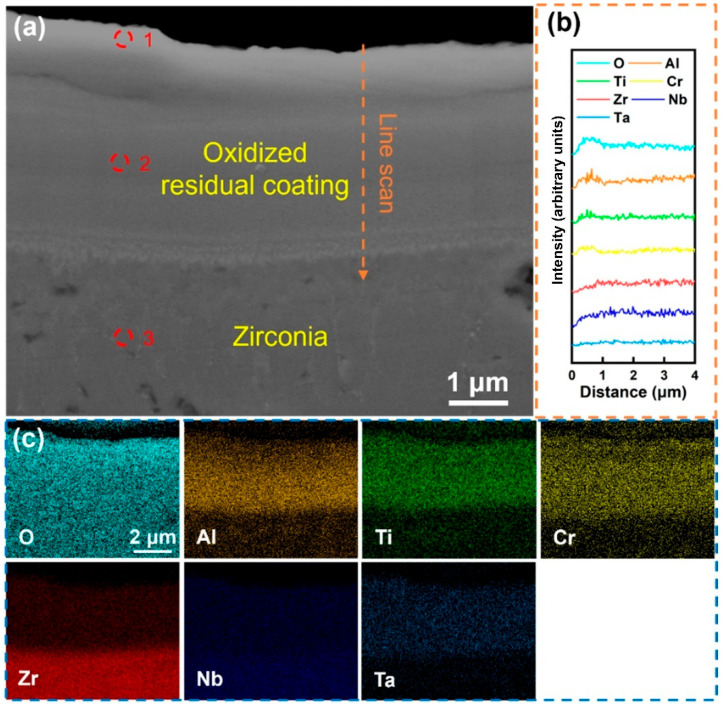
(**a**) Cross-sectional SEM images of the Al-17 coating after holding it in steam at 1000 °C for 60 min; (**b**) EDS line scan of (**a**) at the marked position; (**c**) EDS mapping of (**a**); the corresponding components in (**a**) are shown in Table 6.

The high-temperature water vapor oxidation behavior of the Al-10 coating indicated that the appropriate addition of Al can significantly enhance the oxidation resistance of the coating. However, as the Al content increased further, although the coating remained intact and exhibited strong bonding with the substrate, its oxygen-blocking performance was reduced. Figure 8 shows the cross-sectional morphology and element distribution of the Al-17 coating after being held in a water vapor environment at 1000 °C for 60 min. From the EDS mapping results in Figure 8c, it can be seen that the O content in the substrate has significantly increased, indicating that the coating failed to effectively prevent the diffusion of the O element into the substrate. The distribution of the O element in the coating was relatively obvious. The element analysis at site 1 in Table 6 shows that the coating surface mainly formed a mixed oxide composed of Al_2_O_3_, TiO_2_, and Cr_2_O_3_. The element analysis at site 2 indicates that the coating has been totally oxidized. Although the Al-17 coating can maintain structural integrity, its oxygen-blocking ability is lower than that of the Al-10 coating. The excessive addition of Al may weaken the oxidation resistance of the coating, making O more likely to penetrate and oxidize the substrate (site 3).

**Figure 9:**
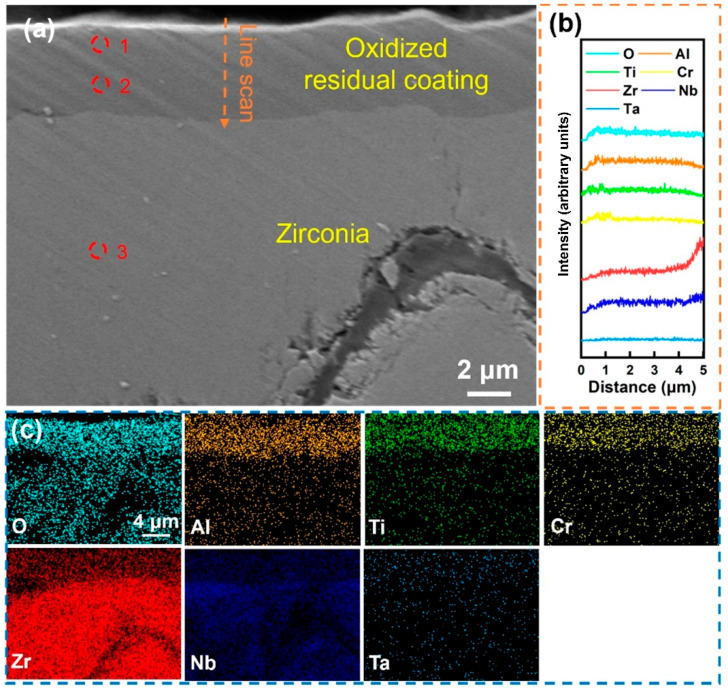
(**a**) Cross-sectional SEM images of the Al-25 coating after holding it in steam at 1000 °C for 60 min; (**b**) EDS line scan of (**a**) at the marked position; (**c**) EDS mapping of (**a**); the corresponding components in (**a**) are shown in Table 7.

Figure 9 shows the cross-sectional morphology and element distribution of the Al-25 coating after being maintained at 1000 °C in a high-temperature water vapor environment for 60 min. After oxidation, the coating still maintains good bonding with the substrate. However, as can be seen from the distribution of the O element in Figure 9c, the substrate has been completely oxidized, and no obvious surface oxide layer has been formed. The line scan results in Figure 9b indicate that there are Al element-enriched regions on the coating surface. The component analysis in Table 7 at site 1 shows that the contents of Al, Ti, and Cr on the coating surface have slightly increased, which is mainly attributed to the surface diffusion effect of elements in the early stage of oxidation. The component analysis at site 2 indicates that the content of the Al element in the coating interior is relatively high, and compared with the deposited-state coating, it has not undergone large-scale diffusion to the surface. However, the component analysis at site 3 shows that the substrate has been completely oxidized, and the coating failed to effectively prevent the diffusion of the O element, ultimately leading to the failure of the coating. Overall, although the Al-25 coating still maintains structural integrity during the high-temperature oxidation process, its oxygen-blocking ability has not been effectively enhanced, and the substrate has suffered severe oxidation, indicating that excessive Al alloying may have an adverse effect on the oxidation resistance of the coating.

**Figure 10:**
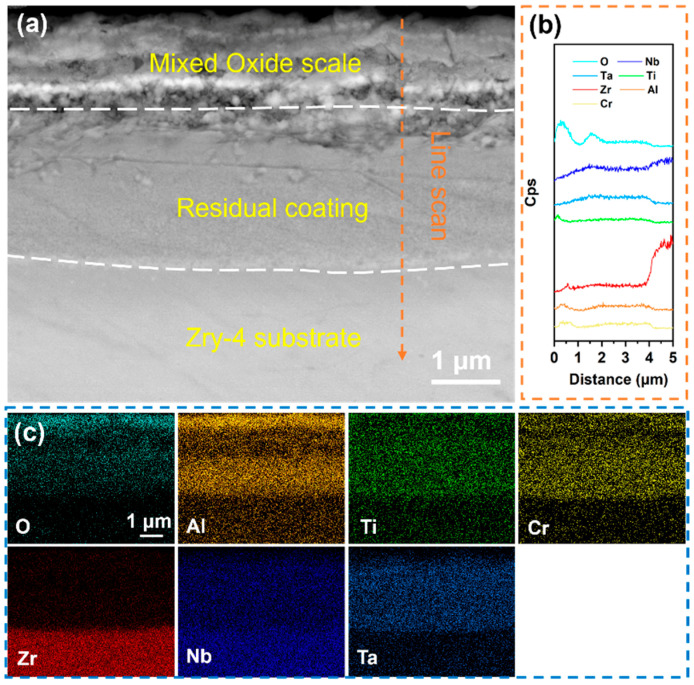
(**a**) Cross-sectional SEM images of the Al-10 coating after holding it in steam at 1100 °C for 30 min; (**b**) EDS line scan of (**a**) at the marked position; (**c**) EDS mapping of (**a**).

Through the high-temperature oxidation experiments of RHEA coatings alloyed with different Al contents, it is known that the addition of active element Al can effectively enhance the oxidation resistance of the coating. However, in order to ensure that the coating can form a stable oxygen-blocking structure during the oxidation process and maintain a good bonding with the substrate to achieve long-term protection, the Al content should be controlled within a reasonable range of 9–14 at.%. To further verify the oxygen-blocking effect of Al on RHEA coatings under high-temperature conditions, a water vapor oxidation experiment at a higher temperature was conducted on the Al-10 coating with better performance. Figure 10a shows the cross-sectional morphology of the Al-10 coating after being held at 1100 °C for 30 min. According to the distribution of the O element in Figure 10b, an oxide layer was formed on the coating surface, which was mainly composed of Al_2_O_3_, TiO_2,_ and Cr_2_O_3_. The experimental results show that the Al-10 coating can still effectively prevent oxygen diffusion under higher temperature environments, demonstrating excellent oxidation resistance, further verifying the positive role of appropriate Al alloying on the oxidation resistance of RHEA coatings.

**Figure 11:**
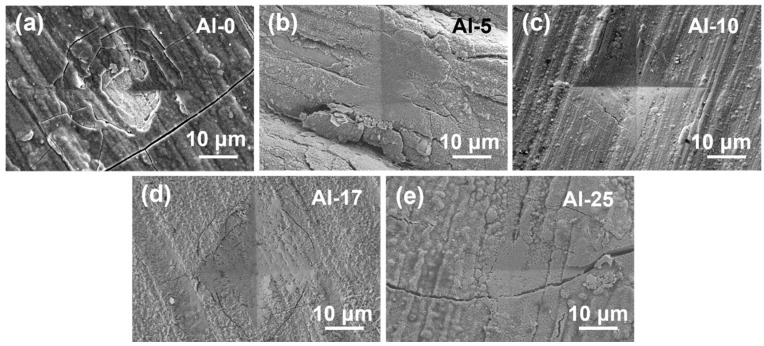
SEM image of the surface indentation of the oxidized state coatings: (**a**) Al-0; (**b**) Al-5; (**c**) Al-10; (**d**) Al-17; (**e**) Al-25.

The refractory high-entropy alloy coating without Al alloying cracked and directly peeled off from the substrate surface, indicating poor bonding with the substrate. At the same time, due to the low plasticity of refractory metal oxides, brittle fracture is prone to occur under external impact, further exacerbating the coating detachment. Al alloying significantly improves coating-substrate bonding, as demonstrated by indentation tests following 1000 °C steam exposure (Figure 11). Unalloyed coatings exhibited catastrophic delamination and brittle fracture under mechanical stress, attributable to the inherent brittleness of refractory metal oxides. In contrast, Al-modified coatings (e.g., Al-5 and Al-10) maintained structural integrity despite localized microcracking. The Al-10 coating, in particular, displayed superior adhesion with minimal internal cracking, a result of its reduced oxygen content and stabilized oxide layer. These findings align with prior studies [38], wherein Al alloying in high-entropy alloys (HEAs) enhanced interfacial cohesion through improved hardness, fracture toughness, and bending resistance. However, excessive Al addition (≥17 at.%) triggered outward crack propagation, albeit without complete structural failure, highlighting Al’s dual role in optimizing adhesion and oxidation resistance. This underscores the necessity of precise Al content regulation to achieve balanced mechanical and anti-oxidative performance in extreme environments.

**Figure 12:**
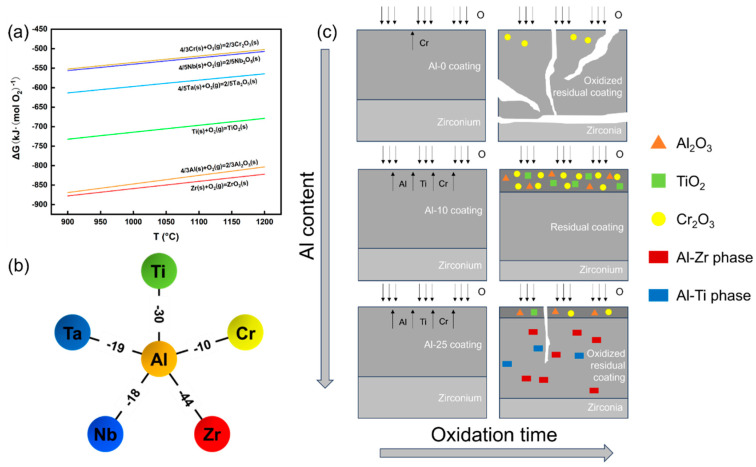
(**a**) Formation free energy of stable oxides of elements in AlTiCrZrNbTa coating at 900–1200 °C; (**b**) heat of mixing of Al with other components in the coating; (**c**) schematic diagram of the oxidation process of AlTiCrZrNbTa coatings with different Al contents during isothermal oxidation in water vapor environment at 1000 °C.

The absence of Al alloying in refractory high-entropy alloy coatings results in fragmented oxidation and substrate exposure due to the inability to form a protective oxide barrier. Al addition promotes surface oxide stability, with the Al-10 coating achieving optimal performance through a dense, continuous Al_2_O_3_-TiO_2_-Cr_2_O_3_ composite layer during high-temperature steam oxidation. Thermodynamic analysis (Figure 12a) reveals that Al_2_O_3_ and TiO_2_ preferentially nucleate due to their lower Gibbs free energy (ΔG) [39,40,41,42,43,44,45,46], while Cr enrichment compensates for its marginally higher ΔG to stabilize Cr_2_O_3_ [47]. Despite ZrO_2_’s thermodynamic stability, its sluggish diffusion kinetics and large atomic size impede surface layer formation. Excessive Al (≥17 at.%) induces Al-Ti/Al-Zr intermetallic phases (Figure 12b), which restrict Al diffusion and disrupt oxide continuity, as explained by the negative mixing enthalpy favoring stable Al-Zr/Ti bonding [48,49]. Concurrently, insufficient Ti/Cr surface enrichment and Al-rich phase accumulation degrade oxidation resistance. These mechanisms underscore the necessity of precise Al content control to balance oxide nucleation kinetics and elemental mobility (Figure 12c), ensuring robust oxygen-blocking performance in extreme environments.

Compared our results with previous studies [50,51]. The literature reports that increasing Al content in alloys enhances the formation of a thicker and more continuous Al_2_O_3_ layer, which protects the substrate from atmospheric oxidation. Our findings differ, as we focus on coatings rather than bulk alloys. In coatings, the compatibility between the residual layer and the oxide layer must be considered. With increasing Al content, thermal expansion mismatch and the accumulation of intermetallics in the residual coating lead to oxide layer cracking and discontinuity. Furthermore, the high oxygen solubility of these intermetallics reduces the overall oxidation resistance of the coating.

## 4. Conclusions

This study systematically evaluates the oxidation resistance of AlTiCrZrNbTa refractory high-entropy alloy (RHEA) coatings on Zry-4 substrates under high-temperature steam environments, with a focus on the critical role of Al alloying. Key findings reveal that Al content profoundly influences coating performance through distinct mechanisms. At an optimal Al concentration of 10 at.%, the coating forms a dense, continuous composite oxide layer (Al_2_O_3_-TiO_2_-Cr_2_O_3_) during oxidation, which effectively suppresses oxygen diffusion into the substrate and maintains robust interfacial adhesion. Thermodynamic analysis confirms the preferential nucleation of Al_2_O_3_ and TiO_2_ due to their low Gibbs free energy, while Cr enrichment enhances oxide layer stability. Mechanical indentation tests further validate the structural integrity of the Al-10 coating, with minimal post-oxidation cracking. Conversely, excessive Al alloying (≥17 at.%) promotes the formation of detrimental Al-Ti and Al-Zr intermetallic phases, which restrict reactive element diffusion and disrupt the continuity of the protective oxide layer, leading to accelerated substrate oxidation. These findings establish a critical Al threshold for balancing oxide layer formation and elemental mobility. The Al-10 coating demonstrates exceptional stability under extreme conditions, highlighting its potential as a durable protective layer for zirconium-based nuclear fuel claddings. This work provides a compositional design framework for optimizing oxidation resistance in RHEA coatings, advancing the development of accident-tolerant fuel systems for enhanced nuclear safety.

## Figures and Tables

**Table 1:** Composition and mixing entropy of AlTiCrZrNbTa coatings.

Sample	Element (at.%)						Δ*S*_mix_
	Ti	Cr	Zr	Nb	Ta	Al	
Al-0	20.49	41.04	14.79	16.36	7.32	-	1.46R
Al-5	18.56	37.48	13.91	15.85	7.74	6.46	1.59R
Al-10	18.41	35.55	11.82	15.78	7.28	11.16	1.64R
Al-17	16.13	33.6	11.53	15.24	6.07	17.43	1.67R
Al-25	18.69	16.42	16.21	17.27	6.01	25.4	1.73R

**Table 2:** Protective evolution of AlTiCrZrNbTa coatings at 1000 °C for 60 min.

Sample	Temperature & Time
	1000 °C 60 min
Al-0	Failed
Al-5	Failed
Al-10	Effective
Al-17	Failed
Al-25	Failed

**Table 3:** Components in the corresponding region in Figure 5.

Point	Element (at.%)					
	O	Ti	Cr	Zr	Nb	Ta
1	82.53	2.92	8.17	2.56	2.44	1.38
2	81.66	3.4	6.36	3.47	3.41	1.70
3	73.65	0.16	0.01	25.78	0.4	0

**Table 4:** Components in the corresponding region in Figure 6.

Point	Element (at.%)						
	O	Al	Ti	Cr	Zr	Nb	Ta
1	68.43	4.78	5.2	14.41	3.78	1.85	1.46
2	65.67	1.58	6.27	12.38	6.01	4.86	3.32
3	64.24	0	0.01	0.07	34.83	0.85	0

**Table 5:** Components in the corresponding region in Figure 7.

Point	Element (at.%)						
	O	Al	Ti	Cr	Zr	Nb	Ta
1	59.85	6.14	10.26	19.28	2.76	1.55	0.16
2	31.42	6.76	9.57	27.95	9.14	10.79	4.37
3	17.35	1.56	0.45	0.81	75.29	4.54	0

**Table 6:** Components in the corresponding region in Figure 8.

Point	Element (at.%)						
	O	Al	Ti	Cr	Zr	Nb	Ta
1	68.63	6.54	6.06	13.59	2.23	1.96	1.17
2	66.68	5.65	4.17	10.32	5.42	5.76	2.45
3	64.48	1.69	0.58	0.32	29.13	0.8	0.31

**Table 7:** Components in the corresponding region in Figure 9.

Point	Element (at.%)						
	O	Al	Ti	Cr	Zr	Nb	Ta
1	82.8	6.18	4.66	3.95	0.6	1.1	0.8
2	77.6	6.5	4.4	2.9	3.6	3.7	1.3
3	76.28	0	0.04	0	23.49	0	0.19

## Data Availability

The original contributions presented in this study are included in the article Further inquiries can be directed to the corresponding author.
